# Micron Scale Spatial Measurement of the O_2_ Gradient Surrounding a Bacterial Biofilm in Real Time

**DOI:** 10.1128/mBio.02536-20

**Published:** 2020-10-20

**Authors:** Alexander D. Klementiev, Zhaoyu Jin, Marvin Whiteley

**Affiliations:** a School of Biological Sciences, Georgia Institute of Technology, Atlanta, Georgia, USA; b Emory-Children’s Cystic Fibrosis Center, Atlanta, Georgia, USA; c Center for Microbial Dynamics and Infection, Georgia Institute of Technology, Atlanta, Georgia, USA; d Center for Electrochemistry, Department of Chemistry, The University of Texas at Austin, Austin, Texas, USA; University of Washington

**Keywords:** biofilm, oxygen, antibiotics, electrochemistry, *Pseudomonas aeruginosa*, antibiotic resistance

## Abstract

Bacteria alter their local chemical environment through both consumption and the production of a variety of molecules, ultimately shaping the local ecology. Molecular oxygen (O_2_) is a key metabolite that affects the physiology and behavior of virtually all bacteria, and its consumption often results in O_2_ gradients within sessile bacterial communities (biofilms). O_2_ plays a critical role in several bacterial phenotypes, including antibiotic tolerance; however, our understanding of O_2_ levels within and surrounding biofilms has been hampered by the difficulties in measuring O_2_ levels in real-time for extended durations and at the micron scale. Here, we developed electrochemical methodology based on scanning electrochemical microscopy to quantify the O_2_ gradients present above a Pseudomonas aeruginosa biofilm. These results reveal that a biofilm produces a hypoxic zone that extends hundreds of microns from the biofilm surface within minutes and that the biofilm consumes O_2_ at a maximum rate. Treating the biofilm with levels of the antibiotic ciprofloxacin that kill 99% of the bacteria did not affect the O_2_ gradient, indicating that the biofilm is highly resilient to antimicrobial treatment in regard to O_2_ consumption.

## Abstract (precis)

O_2_ is a fundamental environmental metabolite that affects all life on earth. While toxic to many microbes and obligately required by others, those that have appropriate physiological responses survive and can even benefit from various levels of O_2_, particularly in biofilm communities. Although most studies have focused on measuring O_2_ within biofilms, little is known about O_2_ gradients surrounding biofilms. Here, we developed electrochemical methodology based on scanning electrochemical microscopy to measure the O_2_ gradients surrounding biofilms in real time on the micron scale. Our results reveal that P. aeruginosa biofilms produce a hypoxic zone that can extend hundreds of microns from the biofilm surface and that this gradient remains even after the addition of antibiotic concentrations that eradicated 99% of viable cells. Our results provide a high resolution of the O_2_ gradients produced by P. aeruginosa biofilms and reveal sustained O_2_ consumption in the presence of antibiotics.

## OBSERVATION

Molecular oxygen (O_2_) is one of the most important molecules dictating bacterial lifestyle and behavior. For organisms capable of tolerating O_2_, it can provide a means to remove excess electrons formed during metabolism. While general fundamentals of O_2_ consumption are well established, the role of O_2_ is complex in bacterial communities, including those associated with human infection, since O_2_ levels vary tremendously based on the infection site and the host response (1–3). In addition, bacteria in many infections grow as sessile communities called biofilms (4), and the three-dimensional structure of these communities can affect O_2_ levels throughout the biofilm.

Previous work has shown that O_2_ gradients within biofilms affect their biology (5, 6). This has prompted an examination of O_2_ levels within and surrounding biofilms. In particular, stagnant biofilms rapidly deplete O_2_ and waste material buildup occurs as a result of mass transport limitation at the surface of biofilms (7). Although it is clear that O_2_ levels are decreased within the biofilm, the levels immediately adjacent to the biofilm surface have not been thoroughly investigated in static biofilms, in part due to the difficulties in robustly measuring O_2_ with high spatial precision (5, 8–13). To address this gap in knowledge, we developed a system to spatially measure O_2_ levels above a microbial biofilm in real time at the micron scale. We chose the facultative anaerobe Pseudomonas aeruginosa strain PA14 for these studies since this opportunistic pathogen preferentially utilizes aerobic respiration (14), and its physiology and behavior are highly influenced by O_2_ availability (14, 15).

**FIG 1:**
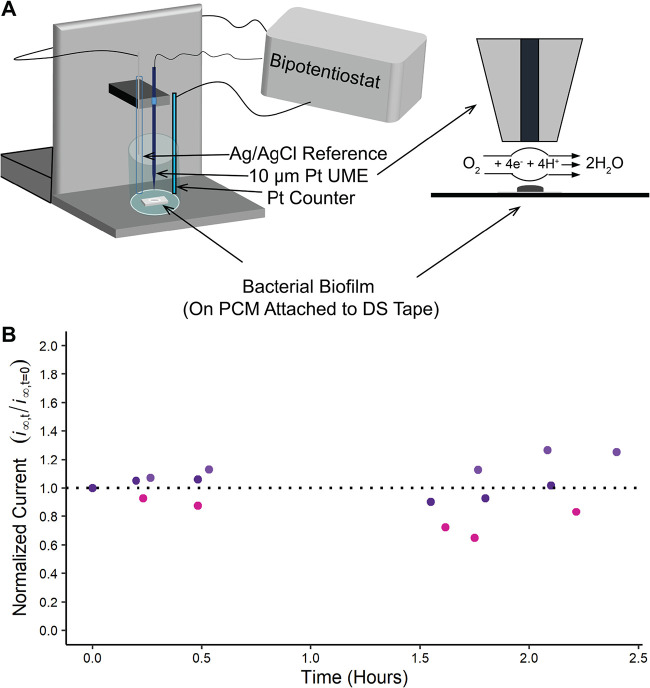
Experimental system and SECM detection of the O_2_ gradient surrounding a P. aeruginosa biofilm. (A) Schematic of SECM setup for measurement of O_2_ gradient surrounding a P. aeruginosa biofilm (left), including a closeup of the SECM cell and O_2_ reduction reaction at the UME tip (right). (B) The platinized UME continuously monitors bulk O_2_ levels through measurement of tip current over several hours without loss of sensitivity. The *y* axis (ordinate) is the ratio of the tip current at each time point divided by the tip current at time zero. Each color represent biological replicates. PCM, polycarbonate membrane; DS, double-sided; UME, ultramicroelectrode.

A significant challenge that was overcame is the inherent difficulty with continuously measuring O_2_ over extended time periods. To address this challenge, we developed a system using electrochemical methods to measure O_2_ in real-time with micron-scale spatial resolution (Fig. 1A). O_2_ can be detected electrochemically through a four-electron reduction on a platinum ultramicroelectrode (UME) (Fig. 1A) (16). However, platinum UMEs readily deactivate which leads to long wait times for O_2_ current stabilization and sub-nA current (see Fig. S1 in the supplemental material). To address this challenge, we optimized a platinization protocol that coats the UME surface with platinum particles that actively reduce O_2_ while avoiding severe changes in the geometry of the UME surface (see Fig. S2). Importantly, our platinum UMEs had a higher electroactive area and could continuously monitor O_2_ levels over several hours without loss of sensitivity (Fig. 1B). This is especially important because the current measured in bulk was approximated to be 205 μM; since current measured is directly proportional to O_2_ concentration, stability ensures accurate O_2_ measurement despite each platinized UME used only once per experiment and having slightly variable degrees of platinization or size after polishing.

10.1128/mBio.02536-20.2 FIG S1 Unplatinized platinum UME measuring O_2_. In contrast to a platinized UME, current measured using an unplatinized platinum UME resulted in low current and a slow decrease in current over time due to fouling. Download FIG S1, PDF file, 0.1 MB. Copyright © 2020 Klementiev et al. 2020 Klementiev et al. This content is distributed under the terms of the Creative Commons Attribution 4.0 International license.

10.1128/mBio.02536-20.3 FIG S2 Electroactive surface area and roughness increases with platinization. (Above) Electroactive area was measured using 0.1 M H_2_SO_4_. Geometric surface area was measured using 1 mM FcMeOH. Rough_F_ = Electroactive Surface Area AFTER/Geometric Surface Area AFTER. Rough_I_ = Electroactive Surface Area BEFORE/Geometric Surface Area BEFORE. “BEFORE” and “AFTER” refer to before and after platinization. (Below) Representative cyclic voltammogram of platinum UME in 0.1 M H_2_SO_4_ for 100 cycles each; blue corresponding to before platinization and yellow after platinization. Download FIG S2, PDF file, 0.1 MB. Copyright © 2020 Klementiev et al. 2020 Klementiev et al. This content is distributed under the terms of the Creative Commons Attribution 4.0 International license.

We next sought to measure O_2_ levels surrounding a P. aeruginosa biofilm using scanning electrochemical microscopy (SECM). The P. aeruginosa strain chosen for this work (*fliC9*::MrT7) (17) has an inactivated flagellar motor protein, rendering the strain unable to leave the biofilm via swimming motility. Biofilms of the P. aeruginosa *fliC* mutant were formed on polycarbonate membranes as previously described for electrochemical studies (18). Membrane biofilms were grown for 8 h on Todd Hewitt broth (THB) agar, yielding an ∼3-mm-diameter nascent biofilm containing ∼4 × 10^7^ bacteria (see Fig. S3). These biofilms contain fewer cells than those used in previous studies (5, 8) focused on O_2_ consumption to better mimic biofilms observed in human infections. After formation, the membrane containing the biofilm was removed from the agar plate and attached to the bottom of a glass vial using double-sided tape and covered with ∼5 ml of morpholinepropanesulfonic acid (MOPS)-glucose minimal medium.

10.1128/mBio.02536-20.4 FIG S3 Image of biofilm (arrow) used in these studies. The average biofilm diameter was 2.94 mm ± 0.24 mm (mean ± the standard deviation, *n* = 48). The vial measures approximately 20 mm (inner diameter) by 25 mm (height) with ∼5 ml of MOPS-glucose minimal media corresponding to a level ∼15 mm above the biofilm surface. Download FIG S3, PDF file, 0.04 MB. Copyright © 2020 Klementiev et al. 2020 Klementiev et al. This content is distributed under the terms of the Creative Commons Attribution 4.0 International license.

**FIG 2:**
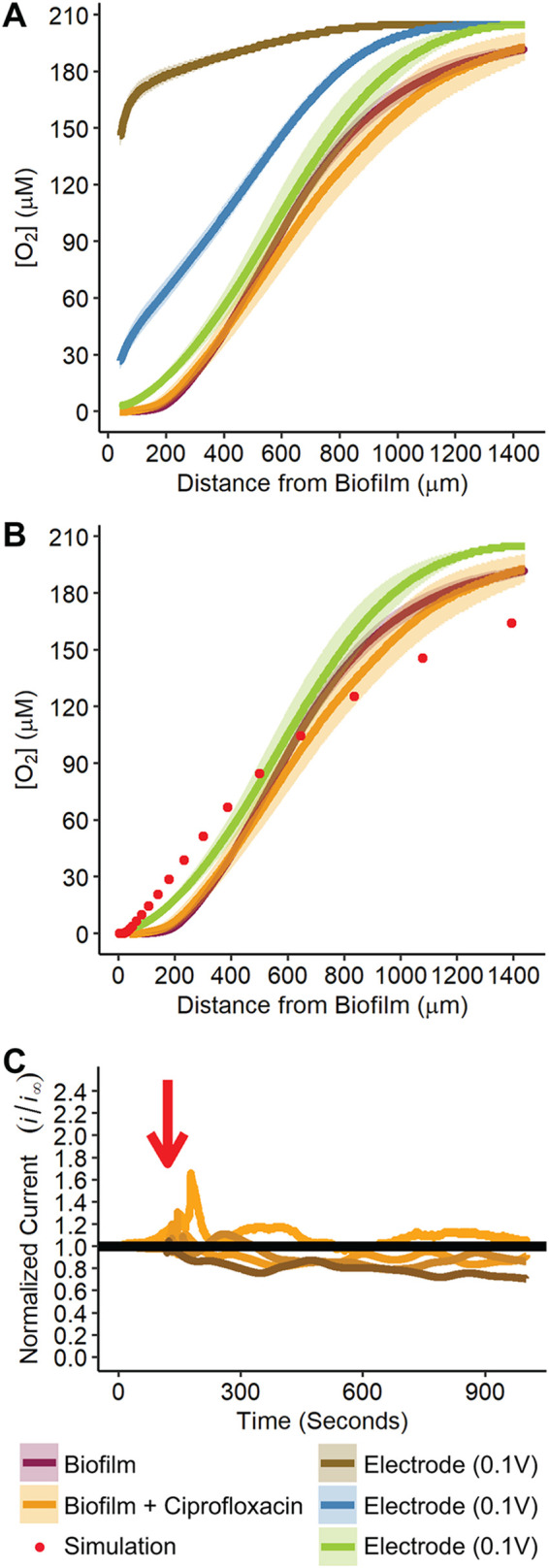
P. aeruginosa rapidly produce O_2_ gradients that are resilient to antibiotic treatment. (A) O_2_ gradients above the surface of P. aeruginosa biofilms, P. aeruginosa biofilms treated with ciprofloxacin, and for reference a 3-mm platinum electrode poised at 0, 0.1, and −0.5 V versus Ag/AgCl (different electrode potentials correspond to various O_2_ consumption rates). *n* = 4 biological replicates for Electrode 0.1 V, Electrode 0 V, Electrode −0.5 V, and Biofilm + Ciprofloxacin, and *n* = 16 biological replicates for biofilm. For all O_2_ gradients, shading represents one standard deviation from the mean (solid line). (B) Digital simulation (red circles) to estimate O_2_ consumption rates of the biofilm. The model was solved by Comsol Multiphysics (5.3a; COMSOL, Inc., Burlington, MA) using the electrochemical analysis module in two-dimensional axial symmetry using stationary conditions with a parametric sweep of the “d” or distance between UME tip and substrate (Fig. S5, and detailed in Text S1). (C) Changes in O_2_ concentration 600 μm above a biofilm measured as a response to ciprofloxacin treatment. At 120 s, the first dose of 20 μg/ml ciprofloxacin was added (designated by red arrow). Each line represents a biological replicate. The *y* axis (ordinate) is the ratio of the tip current at each time point divided by current measured before ciprofloxacin addition (i.e., a value of 1 indicates no change in current after ciprofloxacin addition). There were changes immediately after ciprofloxacin addition (peaks at red arrow), likely a result of the mixing caused by addition of ciprofloxacin to the growth media above the biofilm. Importantly, the current quickly stabilized.

To measure O_2_ levels above the biofilm, a 10-μm-diameter platinized UME was approached to 40 μm above the biofilm surface using ferrocenyl methyl trimethylammonium (FcMTMA^+^; the toxicity and stability are assessed in Text S1 in the supplemental material) as the redox mediator (Fig. S4 and S5) using SECM (19). The UME tip was then poised at −0.5 V (O_2_ reduction potential), with a wait time of 5 min; afterward, the UME was retracted at 6 μm/s while continually measuring O_2_ until bulk O_2_ levels were detected, ∼1,400 μm above the biofilm surface (the O_2_ gradient calculations are detailed in Text S1). The O_2_ levels above the biofilm resembled a sigmoidal curve with no O_2_ detectable until ∼200 μm above the biofilm (Fig. 2A; see also Fig. S6). Assuming a 10-pA minimal background current, the detection limit of the UME is ∼1 μM.

10.1128/mBio.02536-20.1 TEXT S1 Supplemental methods. Download Text S1, PDF file, 0.2 MB. Copyright © 2020 Klementiev et al. 2020 Klementiev et al. This content is distributed under the terms of the Creative Commons Attribution 4.0 International license.

10.1128/mBio.02536-20.5 FIG S4 FcMTMA^+^ does not influence the growth rate of P. aeruginosa. (Above) Growth curve of P. aeruginosa PA14 *fliC9*::MrT7 in MOPS-glucose. Blue points represent growth without FcMTMA^+^ and orange points represent growth with FcMTMA^+^. Experiments represent biological triplicates. (Below) Electrochemical measurements done in tandem with growth experiments to observe changes in FcMTMA^+^ signal. Growth kinetics: data points from 1.5 to 6 h were plotted on a semilog graph and the following equation (X = X_0_e^kt^) was used to determine the growth rate constant *k* (s^−1^) pA is picoamps, and Pt is platinum. Download FIG S4, PDF file, 0.1 MB. Copyright © 2020 Klementiev et al. 2020 Klementiev et al. This content is distributed under the terms of the Creative Commons Attribution 4.0 International license.

10.1128/mBio.02536-20.6 FIG S5 Approach curve to biofilm surface. (A) To determine the distance the UME tip was from the biofilm surface, the UME was first approached to confirm it was near the surface as represented by the orange line. (The inlaid cyclic voltammogram corresponds to FcMTMA^+^ oxidation. We selected +0.5 V for approach curves because it was at a limiting current potential.) The UME was then retracted, and a finer approach was done to 95% of the current. Fit to a mathematical expression, a 95% current decrease corresponds to approximately L = *d/a* = 7.17, where *d* is the distance between the UME tip and the biofilm surface), and *a* is the tip radius (9). (B) With a tip radius (*a*) of 5 μm, this corresponds to a distance (*d*) of ∼40 μm from the biofilm surface. Download FIG S5, PDF file, 0.3 MB. Copyright © 2020 Klementiev et al. 2020 Klementiev et al. This content is distributed under the terms of the Creative Commons Attribution 4.0 International license.

10.1128/mBio.02536-20.7 FIG S6 Calculating O_2_ gradients. The *y* axis (ordinate) is the ratio of the tip current found in bulk (raw current averaged between points at ∼1,400 μm to ∼1,300 μm) divided by current measured at each point. Measured current was converted to gradients by multiplying this ratio (obtained in this graph) by the concentration of O_2_ in bulk (205 μM) to produce Fig. 2. Three biological replicates of all replicates are shown; gold colors represent O_2_ gradients before ciprofloxacin, and blue colors represent O_2_ gradients after ciprofloxacin was added. The red line was added to represent the limit of detection of an unplatinized UME and the limit we generously set for all UMEs. While platinization increased the sensitivity for all UMEs, we observed that this sensitivity limit did not fluctuate drastically. Download FIG S6, PDF file, 0.2 MB. Copyright © 2020 Klementiev et al. 2020 Klementiev et al. This content is distributed under the terms of the Creative Commons Attribution 4.0 International license.

For comparison, we created an O_2_ gradient without a biofilm using a platinum electrode the same size as the biofilm as the SECM substrate. The 3-mm platinum electrode was held at three potentials (0.1, 0, and −0.5 V versus Ag/AgCl) for 5 min, and then the O_2_ gradient was measured as described for the biofilm (detailed in Text S1). The biofilm O_2_ gradient was similar to the −0.5 V poised electrode gradient, which is the potential at which O_2_ reduction is mass transport limited at the surface of the electrode. The biofilm O_2_ gradient was distinct from the other potentials at which O_2_ was being consumed at a submaximal rate (i.e., limited in part by kinetics and not predominantly by mass transport). Using Comsol Multiphysics to digitally simulate O_2_ consumption (Fig. 2B; see also Fig. S7), we approximated the flux of O_2_ at the surface of the biofilm to be 8.2 × 10^−7 ^mol/cm^2^/s (detailed in Text S1). Assuming each cell has a dimension of 1.5 μm × 0.8 μm, 9.8 × 10^−15 ^mol/s O_2_ or 5.9 × 10^9^ molecules of O_2_ per second were consumed by each bacterium. Collectively, these results reveal that P. aeruginosa biofilms produce a hypoxic zone that can extend hundreds of microns from the biofilm surface within minutes, and the biofilm consumes O_2_ at a maximum rate.

10.1128/mBio.02536-20.8 FIG S7 Schematic diagram of Comsol Multiphysics model depicting key features. Simulation using Comsol to determine the O_2_ consumption rate. Download FIG S7, PDF file, 0.03 MB. Copyright © 2020 Klementiev et al. 2020 Klementiev et al. This content is distributed under the terms of the Creative Commons Attribution 4.0 International license.

To assess the effect of antibiotic treatment on biofilm O_2_ consumption, we treated our biofilms with 400 times the MIC of the antibiotic ciprofloxacin (40 μg/ml) and then measured the O_2_ gradient above the biofilm. We first confirmed that ciprofloxacin does not interfere with the electrochemical signal for O_2_ quantification (Fig. S8; see also Text S1). Ciprofloxacin treatment of the biofilm was performed by initially adding 20 μg/ml ciprofloxacin and measuring the O_2_ response 1.5 h after submersion in MOPS-glucose (Fig. 2C). After we observed no immediate change in signal, we treated the biofilm with another 20 μg/ml. After addition of the second dose of ciprofloxacin, the O_2_ gradient was measured for 50 min with no observable change in the O_2_ gradient, a total of 1 h and 35 min after ciprofloxacin was first added. Despite the fact that addition of ciprofloxacin reduced the number of viable bacteria in the biofilm by 100-fold to ∼2.4 × 10^5^ bacteria, there was no change in the O_2_ gradient or O_2_ consumption rates (Fig. 2A and B).

10.1128/mBio.02536-20.9 FIG S8 Addition of ciprofloxacin to MOPS-glucose does not interfere with O_2_ measurement. Steady-state voltammograms recorded within the potential window between +0.6 V and −0.5 V in the presence of ambient O_2_ (red), ambient O_2_ and 200 μg/ml ciprofloxacin (green), and O_2_ purged solution containing 200 μg/ml ciprofloxacin (blue). Representative data from triplicate experiments are shown. Download FIG S8, PDF file, 0.2 MB. Copyright © 2020 Klementiev et al. 2020 Klementiev et al. This content is distributed under the terms of the Creative Commons Attribution 4.0 International license.

While prior work has primarily measured bulk O_2_ at the biofilm/air interface, we show that, in contrast, at a stagnant biofilm/liquid interface a hypoxic region forms several hundred microns above the biofilm surface. Containing only ∼4 × 10^7^ bacteria, our biofilms consumed O_2_ at maximum rates and continued to do so despite 99% killing by ciprofloxacin. These data corroborate similar findings that bulk respiratory activity and carbon consumption persists despite antibiotic exposure (20, 21). Given this high O_2_ consumption rate and the observation that biofilms of this size exist in human implant/catheter infections (22), we propose that biofilms are capable of rapidly depleting local O_2_ in chronic infections even during antibiotic challenge. Ultimately, the experimental system developed in this work provides a valuable framework for studying biofilm O_2_ consumption.

## MATERIALS AND METHODS

### Instrumentation.

Initial electrochemistry experiments were performed using a BioLogic SECM (model M470). Biofilm experiments measuring O_2_ gradients by scanning distances were done using a CHI model 920D scanning electrochemical microscope (CH Instruments). For all experiments, a three-electrode setup was used. This consisted of a 10-μm-diameter platinum UME (working electrode), Ag|AgCl|Saturated KCl (reference electrode to which all potentials are referred to in all experiments), and platinum wire (counter electrode). An in-depth protocol for UME fabrication may be found elsewhere (19).

### Ultramicroelectrode fabrication and SECM cell setup.

An in-depth protocol for UME fabrication may be found elsewhere (19). Briefly, platinum (99.9% purity) wire, 10-μm diameter, temper: hard (Goodfellow Metals, Cambridge, United Kingdom; product PT005107) was used for the preparation of the SECM UME tip. The metal wire was heat sealed with a heating coil under vacuum in a glass capillary. The tip was sharpened to an RG of ∼10, where RG is the ratio of the glass diameter to wire diameter. Prior to electrochemical experiments, UMEs were sonicated in a water bath for 30 s. Platinization significantly alters the surface and UMEs were seldom repolished and replatinized for reuse.

### Bacterial strain culture and preparation.

P. aeruginosa (PA14) *fliC9*::MrT7 mutant was obtained from a PA14 nonredundant transposon insertion mutant set (http://ausubellab.mgh.harvard.edu/cgi-bin/pa14/home.cgi) (17). Biofilms were grown in THB agar for 8 h at 37°C, at which point an ∼3-mm-diameter biofilm formed before transfer to the SECM cell. All SECM experiments were performed using MOPS minimal media (23) containing 20 mM glucose. CFU were enumerated at the end of experimentation by removing media above the biofilm, substantially vortexing the biofilm off the polycarbonate membrane, and plating on THB agar plates overnight at 37°C.

### Platinizing UMEs.

Handmade 10 μm platinum UMEs (as described above) were sonicated in water, acetone, and water. A modified protocol for platinizing UMEs was used that may be found elsewhere (24) with an adjusted recipe for the platinization solution containing 0.250 ml of H_2_PtCl_6_ and 0.4 mg of Pb(NO_3_)_2_ up to a final volume of 7.36 ml in 1× phosphate-buffered saline (pH 7.4). Geometric and electroactive effects on the UME surface resulting from platinization were measured to confirm the stability and reproducibility of platinization. An in-depth review of platinizing electrodes can be found elsewhere (25).

### Measuring O_2_.

PA14 tn::*fliC* biofilms were grown as described above. After 8 h growth, the polycarbonate membrane was removed and attached to the bottom of a custom glass vial using double sided tape. UMEs were cycled in platinizing solution (same as above) from 0.2 V to −0.3 V versus Ag/AgCl at 100 mV/s until the maximum limiting current increased ∼1.2× for FcMTMA^+^ oxidation (the synthesis is detailed in Text S1). After platinization and ensuring proper geometric area of the UMEs, 1 mM FcMTMA^+^ was added to MOPS-glucose media, and approximately 5 ml was added to the vial containing the biofilm. A three-electrode setup using a platinum wire counter, and Ag/AgCl reference electrodes were connected. Platinized UMEs were precisely positioned with micron-scale accuracy using SECM. SECM positions UMEs at defined distances from the biofilm surface using an electroactive mediator while observing tip current changes as a function of distance. For this work, we chose the electroactive mediator FcMTMA^+^ since it is neither consumed by nor is toxic to P. aeruginosa. UMEs were poised at 0.5 V to oxidize FcMTMA^+^, approached within ∼40 μm above the surface (within the hindered diffusion region corresponding to a decrease in signal to ∼95% limiting current), and then poised at −0.5 V and retracted at 6 μm/s to measure O_2_ gradients. For antibiotic chronoamperometry curves, UMEs were positioned first approximately 600 μm for the first addition of ciprofloxacin or control, approximately 1 h and 30 min elapsed after MOPS-glucose was added over the biofilm. Ciprofloxacin was added slowly during this time at ∼7.5 mm above the biofilm, and the stage was attached to the vial containing the biofilm was rotated 10 times in a circular motion immediately after addition. The UME was then approached approximately 300 μm above the biofilm for the second addition of ciprofloxacin, approximately 2 h elapsed after MOPS-glucose was added over the biofilm. Ciprofloxacin or control was then added quickly at ∼7.5 mm above the biofilm. For both additions, a 2-min window was given before antibiotics were added to the media and current was measured for a minimum of 1,000 s in total. O_2_ concentration gradients were immediately measured in triplicate during each biological replicate after the second addition of antibiotics to determine the O_2_ consumption rates.
