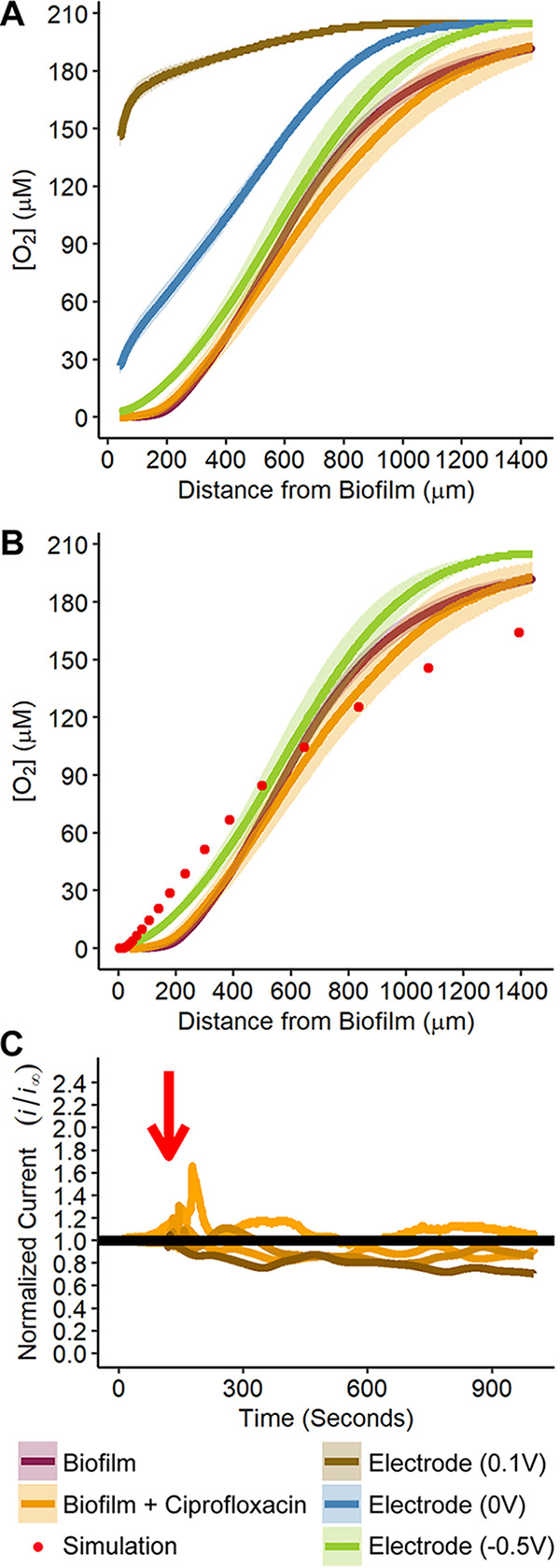# Erratum for Klementiev et al., “Micron Scale Spatial Measurement of the O_2_ Gradient Surrounding a Bacterial Biofilm in Real Time”

**DOI:** 10.1128/mbio.00803-22

**Published:** 2022-04-25

**Authors:** Alexander D. Klementiev, Zhaoyu Jin, Marvin Whiteley

**Affiliations:** a School of Biological Sciences, Georgia Institute of Technology, Atlanta, Georgia, USA; b Emory-Children’s Cystic Fibrosis Center, Atlanta, Georgia, USA; c Center for Microbial Dynamics and Infection, Georgia Institute of Technology, Atlanta, Georgia, USA; d Center for Electrochemistry, Department of Chemistry, The University of Texas at Austin, Austin, Texas, USA

## ERRATUM

Volume 11, no. 5, e02536-20, 2020, https://doi.org/10.1128/mBio.02536-20. In Fig. 2, the legend previously had all electrode potentials labeled “Electrode (0.1V).” The correctly labeled electrode potentials now appear in the figure below.

**Figure fig1:**