# Supplementary figures and images for: Micron Scale Spatial Measurement of the O2 Gradient Surrounding a Bacterial Biofilm in Real Time
Source: mBio. 2020 Oct 20;11(5):e02536-20. doi: 10.1128/mBio.02536-20 (PMC7587442; doi:10.1128/mBio.02536-20)

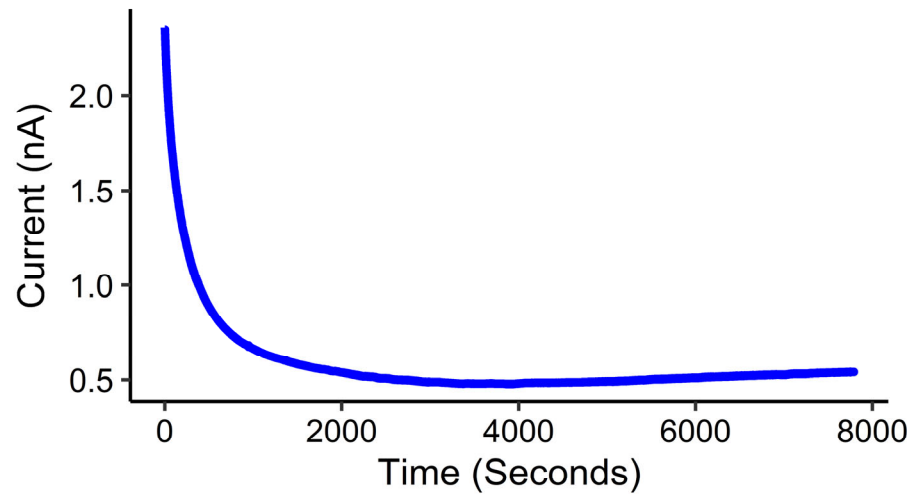

Supplement: FIG S1 [file mBio.02536-20-sf001.pdf]

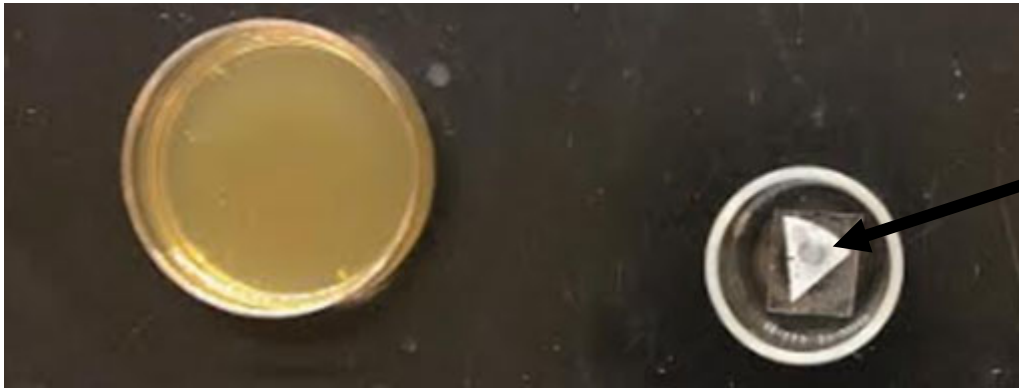

Supplement: FIG S3 [file mBio.02536-20-sf003.pdf]

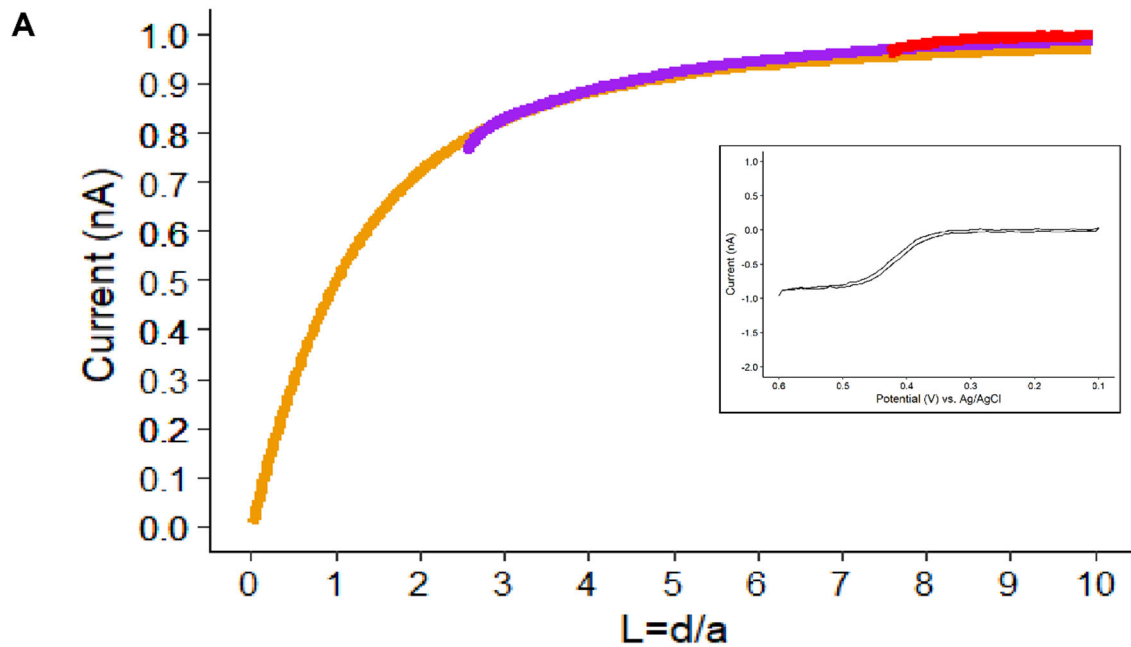

— Mathematical Expression — AC 75% — AC 95%

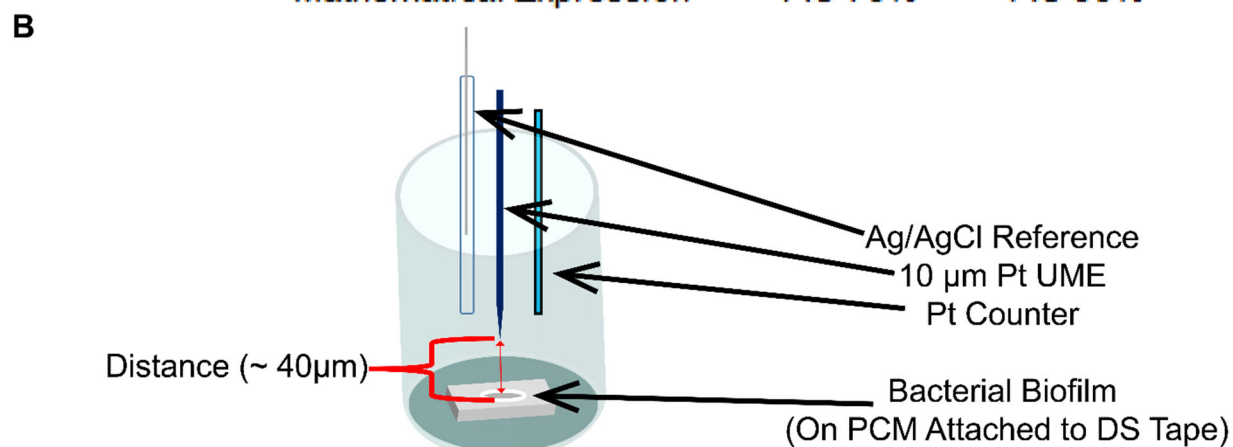

Supplement: FIG S5 [file mBio.02536-20-sf005.pdf]

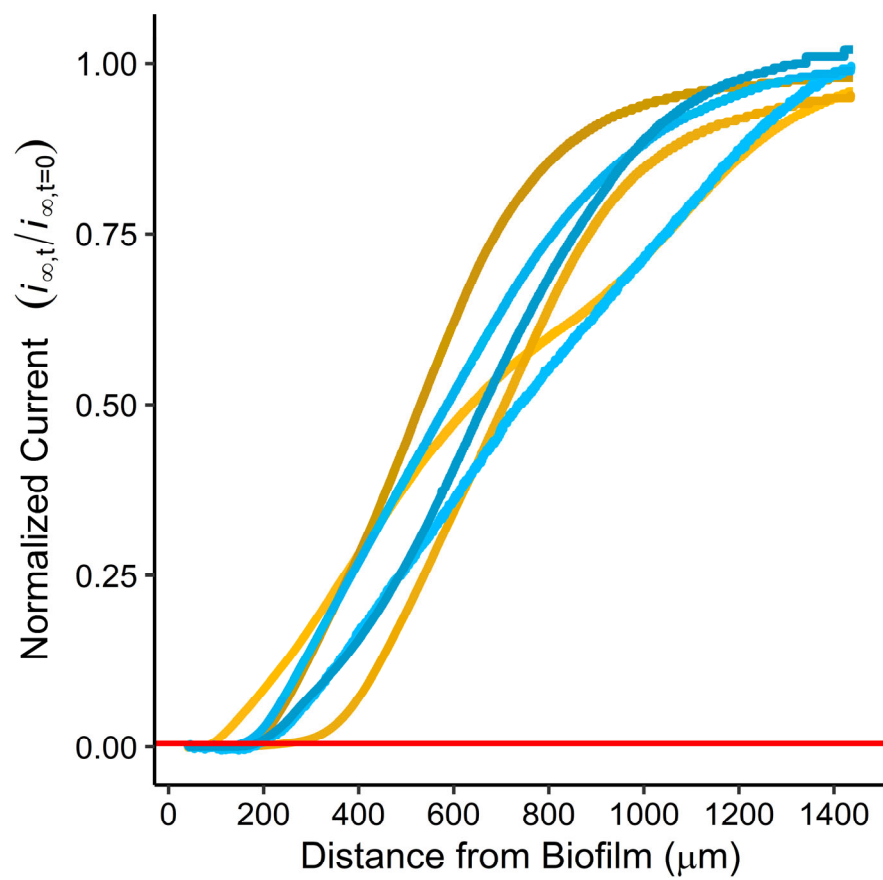

Supplement: FIG S6 [file mBio.02536-20-sf006.pdf]

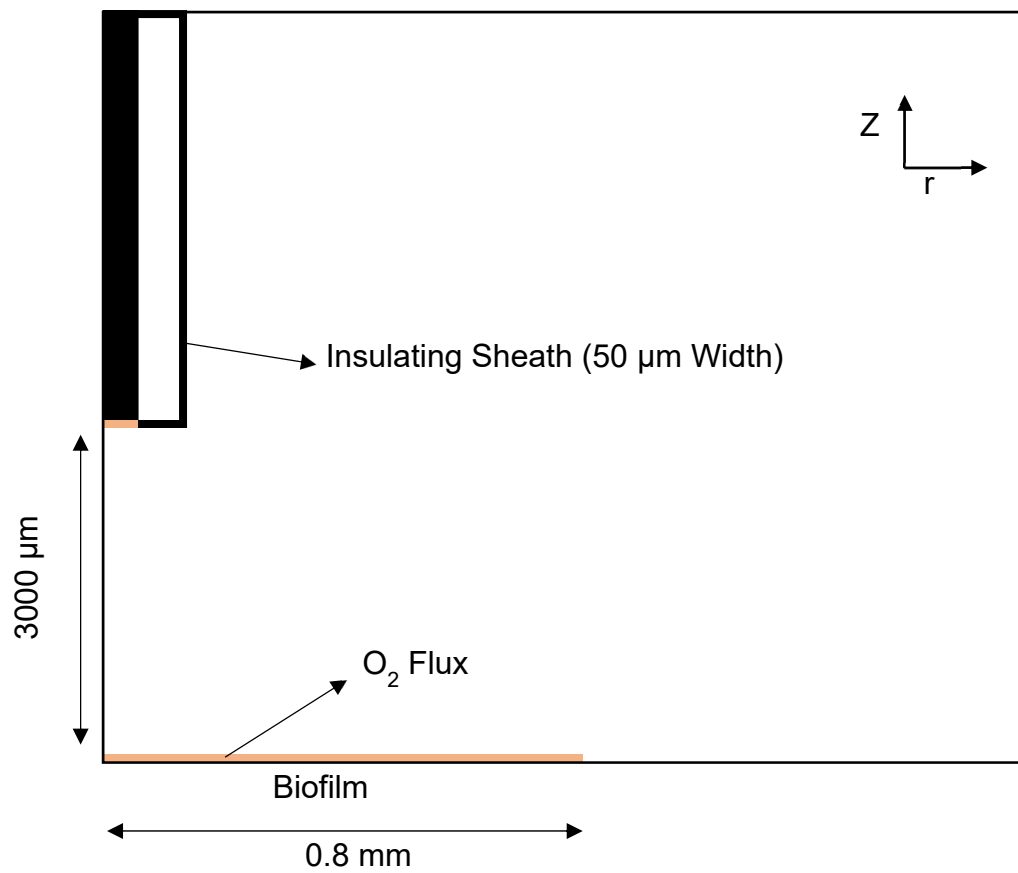

Supplement: FIG S7 [file mBio.02536-20-sf007.pdf]

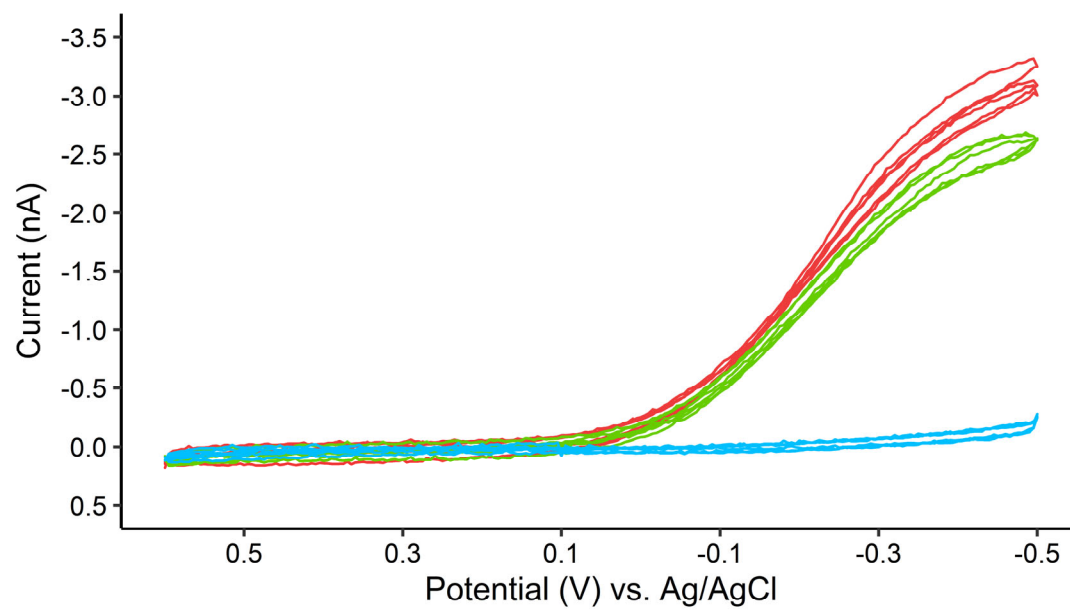

■  $O_2$  ■  $O_2$  and 200  $\mu\text{g/mL}$  Ciprofloxacin ■ 200  $\mu\text{g/mL}$  Ciprofloxacin

Supplement: FIG S8 [file mBio.02536-20-sf008.pdf]
