# Supplementary material for: Micron Scale Spatial Measurement of the O2 Gradient Surrounding a Bacterial Biofilm in Real Time
Source: mBio. 2020 Oct 20;11(5):e02536-20. doi: 10.1128/mBio.02536-20 (PMC7587442; doi:10.1128/mBio.02536-20)

Replicate	Geometric Surface Area BEFORE (Cm^2)	Geometric Surface Area AFTER (Cm^2)	Electroactive Surface Area BEFORE (Cm^2)	Electroactive Surface Area AFTER (Cm^2)	$Rough_F/Rough_I$
**1**	1.65×10^{-6}	2.19×10^{-6}	1.92×10^{-6}	1.41×10^{-5}	5.53
**2**	1.76×10^{-6}	2.01×10^{-6}	7.60×10^{-7}	6.73×10^{-6}	7.72
**3**	1.22×10^{-6}	1.36×10^{-6}	2.23×10^{-6}	1.79×10^{-5}	7.2

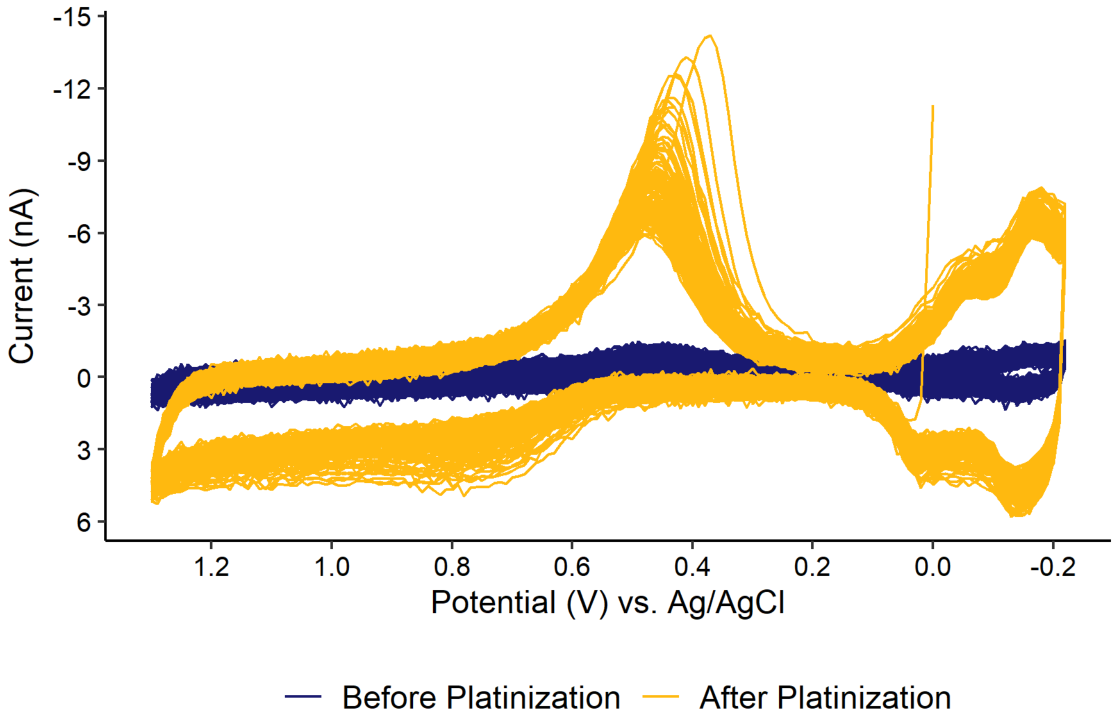

Supplement: FIG S2 [file mBio.02536-20-sf002.pdf]
