# Supplementary material for: Micron Scale Spatial Measurement of the O2 Gradient Surrounding a Bacterial Biofilm in Real Time
Source: mBio. 2020 Oct 20;11(5):e02536-20. doi: 10.1128/mBio.02536-20 (PMC7587442; doi:10.1128/mBio.02536-20)
Supplement: TEXT S1 [file mBio.02536-20-s0001.pdf]

Materials and Methods

General synthesis of Ferrocenyl methyl trimethylammonium ($FcMTMA^+$) by metathesis. In a 70% ethanol washed glass beaker, (ferrocenylmethyl)trimethylammonium iodide was dissolved in minimal amounts of water (0.77 g to ~37 mL water) and sonicated. In another beaker, sodium perchlorate solution (0.245 g dissolved in ~ 2 mL water) was sonicated to dissolve completely. Sodium perchlorate was added to the (ferrocenylmethyl)trimethylammonium iodide solution dropwise and heated while stirring until the solution became clear brown. Heating and stirring continued until the solution evaporated to ~70% the original volume and cooled until visible crystal formation was observed. The solution was frozen at -20°C for 15 minutes in a 50 mL conical after which the top green layer was removed, leaving the $FcMTMA^+$ crystals. The $FcMTMA^+$ was then washed with 40 mL water, and the $FcMTMA^+$ crystals were returned to the beaker and heated with stirring to dissolve. The dissolved $FcMTMA^+$ crystals were cooled at room temperature for five minutes followed by freezing at -20°C for ten minutes then transferred to a 50 mL conical and centrifuged at 4000 x g for two minutes at 10°C to pellet the crystals. The aqueous layer above the crystals was removed, and the crystals washed 4 times with 27 mL of water then vacuum dried. A similar synthesis of $FcMTMA^+$ is described elsewhere (1, 2).

Assessing toxicity and stability of $FcMTMA^+$. To test the toxicity and stability of $FcMTMA^+$, *P. aeruginosa* PA14 *fliC9*::MrT7 was grown overnight in THB broth supplemented with 30 µg/mL of gentamicin. The overnight culture was diluted to an OD_{600} = 0.05 and allowed to grow to mid-logarithmic phase (OD_{600} ~ 0.5) in fresh THB. 1 mL of cell culture was then washed in MOPS-glucose with or without 1 mM $FcMTMA^+$. Cells were then resuspended to an OD_{600} = 0.05 in MOPS-glucose with or without 1 mM $FcMTMA^+$, and the optical density was measured at regular time intervals to assess the effect of $FcMTMA^+$ on *P. aeruginosa* growth. To examine the stability of $FcMTMA^+$, at 0, 3, and 12 hours, 500 µL of the bacterial culture was removed, filtered through a 0.2 µm filter to remove cells, and combined with 200 µL 10X PBS and the current for $FcMTMA^+$ at the limiting current potential was measured. All growth curves represent biological triplicate experiments performed on different days, using different UMEs, and different stocks of $FcMTMA^+$ prepared on different days.

Calculating O_2 Concentration Gradients. The initial concentration of O_2 was assumed to be 205 µM (given a *S*, solubility of O_2 in culture media, of 1.29×10^{-3} mol/m^{3*}mmHg (3, 4) and partial pressure of O_2 to be 158.8 mmHg) with a diffusion coefficient of 2.08×10^{-9} (m^2/s) (5). Platinum UMEs were platinized corresponding to at most an increase in 1.2X the limiting current of ferrocene oxidation. This is because the geometry of a disc-shaped UME follows the following equation for ferrocene oxidation on the surface: $i = 4nfCDa$, whereas a hemisphere-shape resulting from overplatinization would have an equation of: $i = 2\pi nfCDa$. Dividing the latter by the former yields ~1.57X larger measured current, therefore an ~1.2X current increase would indicate that the UME is predominantly more disc-shaped. This resulted in an increase in electroactive area of ~6-7X the original electroactive area. After determining the platinization was reproducibly increasing the electroactive area of the UME while maintaining the geometry disc-like, we proceeded to obtain O_2 gradients. Because platinization results in differences among UME electroactive areas, it was important to consider the limiting current–the current measured in bulk solution. While replicates had similar limiting current in bulk solution that remained stable throughout the experiment–ensuring the electrode was indeed stable for O_2 measurement–the limiting current varied among biological replicates. To account for UME differences observed among biological replicates, we normalized the entire gradient obtained by the limiting current of that biological replicate with the bulk current being normalized to 1. Since the concentration of O_2 is directly proportional to current at the UME (for both a disc-shaped and hemisphere UME), we next multiplied each point by 205 µM to obtain the concentration gradient for each biological replicate individually. Of concern, the lower limit of detection varied since each platinized UME had different levels of sensitivity. We rationalized that the lowest limit of detection must be of an unplatinized UME. Using the equation for a disc shape, $i = 4nfCDa$, that limit was approximated to be ~1 µM. This limit was sufficient to observe gradients up to ~200 µm or less from the biofilm surface. Similar methodology has been used elsewhere (6).

O_2 consumption rates of a 3mm platinum UME. MOPS-glucose with 1 mM ferrocenemethanol was added to a SECM cell with a 3 mm platinum UME as the substrate. A 10 µm diameter platinized platinum tip was placed 40 µm away from the 3 mm platinum UME using a feedback approach curve (coinciding with a 105% increase in current). Next, the substrate was held at various potentials (0.1V, 0V, and -0.5V vs Ag/AgCl) for a minimum of five minutes to allow for the O_2 concentration gradient to develop, and the 10 µm diameter tip was retracted at 6 µm/sec. Between each measurement, a needle and syringe was used to inject approximately 20 mL of air into the system as a means of recalibrating the O_2 homogeneity in solution.

Simulation of O_2 consumption. The simulation modeled the O_2 consumption of a 3 mm platinum UME measured by a 5 µm radius SECM UME using similar geometries found elsewhere (7, 8). The SECM UME (RG = 10) was positioned 3000 µm away from the substrate and held at the diffusion limited potential (-0.5V). The model was solved by Comsol Multiphysics (5.3a, COMSOL Inc., Burlington, MA) using the electrochemical analysis module in 2D axial symmetry using stationary conditions with a parametric sweep of the "d" or distance between UME tip and substrate. An Integral Component Coupling was used to calculate the UME current and post processing of the data included normalizing the current by dividing by

$$I_{tip} = 4nFcDr_{tip}$$

(n =1, F = 96485C/mol, c = 205 µM, D = 2.08×10^{-9} m^2/s, r_{tip} = 5.0 µm).

Since the O_2 concentration gradient of a 3 mm biofilm resembled that of a 3 mm platinum UME held at a potential of -0.5 V, we determined the rate of reaction (following Butler-Volmer kinetics) resulting in the same O_2 gradient as we observed experimentally. Movement of O_2 toward either the working UME or substrate depends on a concentration gradient and therefore Fick's second law of diffusion was used in the simulation given as follows in cylindrical coordinates.

$$\frac{\partial c}{\partial t} = D(\frac{\partial^2 c}{\partial r^2} + \frac{1}{r} + \frac{\partial c}{\partial r} + \frac{\partial^2 c}{\partial z^2})$$

To account for density effects that arise at larger distances, a minimal convective force of $-1 \times 10^{-7}\ m/s$ was applied. Finally, the mesh was adjusted so that it was finer at the boundary of the working and substrate UME and the domain containing O_2 was assumed to have large amounts of supporting electrolytes to mitigate any migration or solution resistance effects. The flux ($mol/m^2/s$) was calculated by multiplying the simulated approximate kinetic rate (4 cm/s) by the concentration of bulk O_2 (205 µM) to obtain a calculated flux of 8.2×10^{-7} $mol/cm^2/sec$. As a control of this simulation, we set the kinetic rate of the substrate to zero and observed pure negative feedback as expected.

Testing the response to ciprofloxacin. Three steady state cyclic voltammetry scans from +0.6V to -0.5V (25 mV/sec) were initially done, and the O_2 reduction reaction peak was observed. Next, the same cyclic voltammetry experiment was performed with 200 µg/mL ciprofloxacin and no observable peak other than the O_2 reduction was observed. O_2 was purged using Argon gas for approximately 15 minutes and the same cyclic voltammetry experiment was performed.

Fig. S1. Unplatinized platinum UME measuring O_2. In contrast to a platinized UME, current measured using an unplatinized platinum UME resulted in low current and a slow decrease in current over time due to fouling.

Fig. S2. Electroactive surface area and roughness increases with platinization. (Above) Electroactive area was measured using 0.1 M H_2SO_4. Geometric surface area was measured using 1 mM FcMeOH. $Rough_F$ = Electroactive Surface Area AFTER/Geometric Surface Area AFTER. $Rough_I$ = Electroactive Surface Area BEFORE/Geometric Surface Area BEFORE. 'BEFORE' and 'AFTER' refer to before and after platinization. (Below) Representative cyclic voltammogram of platinum UME in 0.1 M H_2SO_4 for 100 cycles each; blue corresponding to before platinization and yellow after platinization.

Fig. S3. Image of biofilm (arrow) used in these studies. Average biofilm diameter was 2.94 mm ± 0.24 mm (mean ± standard deviation, n=48). The vial measures approximately 20 mm (inner diameter) by 25 mm (height) with ~5 mL MOPS-glucose minimal media corresponding to a level ~15 mm above the biofilm surface.

Fig. S4. $FcMTMA^+$ does not influence the growth rate of *P. aeruginosa*. (Above) Growth curve of *P. aeruginosa* PA14 *fliC9*::MrT7 in MOPS-glucose. Blue points represent growth without $FcMTMA^+$ and orange points represent growth with $FcMTMA^+$. Experiments represent biological triplicates. (Below) Electrochemical measurements done in tandem with growth experiments to observe changes in $FcMTMA^+$ signal. Growth Kinetics: Data points from 1.5 to 6 hours were plotted on a semilog graph and the following equation ($X = X_0e^{kt}$) was used to determine the growth rate constant k (s^{-1}) pA is picoamps, and Pt is platinum.

Fig. S5. Approach curve to biofilm surface. (A) To determine the distance the UME tip was from the biofilm surface, the UME was first approached to confirm it was near the surface as represented by the orange line. (Inlaid cyclic voltammogram corresponds to $FcMTMA^+$ oxidation. We selected +0.5 V for approach curves because it was at a limiting current potential). Then the UME was retracted and a finer approach was done to 95% of the current. Fit to a mathematical expression, a 95% current decrease corresponds to approximately L=d/a =7.17 where d is the distance between the UME tip and the biofilm surface) and a is the tip radius (9); **(B)** with a tip radius (a) of 5 µm, this corresponds to a distance (d) of ~40 µm from the biofilm surface.

Fig. S6. Calculating O_2 Gradients. The y-axis (ordinate) is the ratio of the tip current found in bulk (raw current averaged between points at ~1400 µm to ~1300 µm) divided by current measured at each point. Measured current was converted to gradients by multiplying this ratio (obtained in this graph) by the concentration of O_2 in bulk (205 µM) to produce **Fig. 2**. Shown are three biological replicates of all replicates, gold colors represent O_2 gradients before ciprofloxacin and blue colors represent O_2 gradients after ciprofloxacin was added. The red line was added to represent the limit of detection of an unplatinized UME and the limit we generously set for all UMEs. While platinization increased sensitivity for all UMEs, we observed this sensitivity limit did not fluctuate drastically.

Fig. S7. Schematic diagram of Comsol Multiphysics model depicting key features. Simulation using Comsol to determine the O_2 consumption rate.

Fig. S8. Addition of ciprofloxacin to MOPS-glucose does not interfere with O_2 measurement. Steady-state voltammograms recorded within the potential window between +0.6V and -0.5V in the presence of ambient O_2 (red), ambient O_2 and 200 µg/mL ciprofloxacin (green), and O_2 purged solution containing 200 µg/mL ciprofloxacin (blue). Representative data of triplicate experiments is shown.

Literature Cited

1. Postlethwaite TA, Samulski ET, & Murray RW (1994) Electrochemical Detection of Anisotropic Probe Diffusion in the Liquid Crystalline Cesium Pentadecafluorooctanoate/D2O System. *Langmuir* 10(7):2064-2067.
2. Kim J*, et al.* (2016) Electrocatalytic Activity of Individual Pt Nanoparticles Studied by Nanoscale Scanning Electrochemical Microscopy. *Journal of the American Chemical Society* 138(27):8560-8568.
3. Lesher-Pérez SC*, et al.* (2017) Dispersible oxygen microsensors map oxygen gradients in three-dimensional cell cultures. *Biomater Sci* 5(10):2106-2113.
4. Morsiani E*, et al.* (2001) Long-term expression of highly differentiated functions by isolated porcine hepatocytes perfused in a radial-flow bioreactor. *Artificial organs* 25(9):740-748.
5. Hung GW & Dinius RH (1972) Diffusivity of oxygen in electrolyte solutions. *Journal of Chemical & Engineering Data* 17(4):449-451.
6. Nebel M, Grützke S, Diab N, Schulte A, & Schuhmann W (2013) Visualization of Oxygen Consumption of Single Living Cells by Scanning Electrochemical Microscopy: The Influence of the Faradaic Tip Reaction. *Angewandte Chemie International Edition* 52(24):6335-6338.
7. Abucayon E*, et al.* (2014) Investigating Catalase Activity Through Hydrogen Peroxide Decomposition by Bacteria Biofilms in Real Time Using Scanning Electrochemical Microscopy. *Analytical Chemistry* 86(1):498-505.
8. Liu X*, et al.* (2011) Real-time mapping of a hydrogen peroxide concentration profile across a polymicrobial bacterial biofilm using scanning electrochemical microscopy. *Proc Natl Acad Sci U S A* 108(7):2668-2673.
9. Bard AJ & Mirkin MV (2012) *Scanning Electrochemical Microscopy* (CRC Press, Boca Raton) 2nd Edition Ed.
