# Supplementary material for: Micron Scale Spatial Measurement of the O2 Gradient Surrounding a Bacterial Biofilm in Real Time
Source: mBio. 2020 Oct 20;11(5):e02536-20. doi: 10.1128/mBio.02536-20 (PMC7587442; doi:10.1128/mBio.02536-20)
Supplement: FIG S4 [file mBio.02536-20-sf004.pdf]

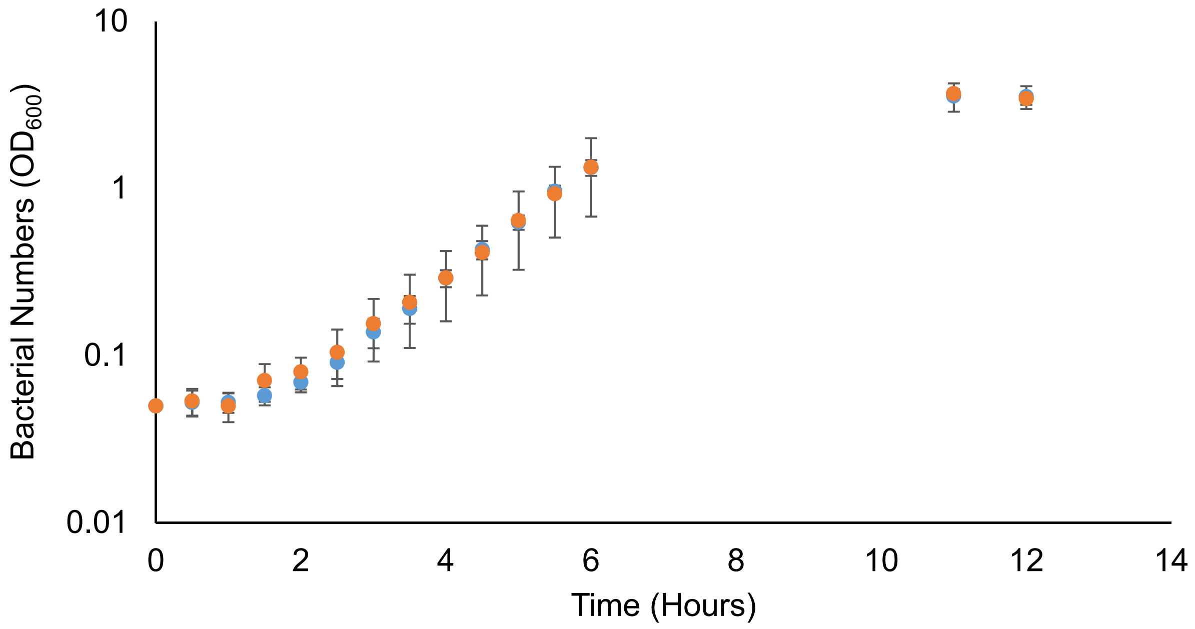

**Replicate**	**Current 0 Hours**	**Current 3 Hours**	**Current 12 Hours**	**Pt UME (μm)**
**1**	7.24 pA	13.03 pA	68.44 pA	10
**2**	-3.34 pA	-5.94 pA	85.25 pA	10
**3**	13.03 pA	63.96 pA	60.75 pA	25

**Replicate**	**Current 0 Hours**	**Current 3 Hours**	**Current 12 Hours**	**mg FcMTMAP**	**Pt UME (μm)**
**1**	966.90 pA	916.47 pA	945.09 pA	5.10 mg	10
**2**	843.84 pA	797.30 pA	871.79 pA	5.01 mg	10
**3**	2273.46 pA	2104.80 pA	2244.00 pA	5.35 mg	25

**Replicate**	***k* without FcMTMAP**	***k* with FcMTMAP**
**1**	0.6777	0.7163
**2**	0.6793	0.5333
**3**	0.7131	0.6739
Average	*0.69*	*0.6492*
